# Down-regulation of GADD45A enhances chemosensitivity in melanoma

**DOI:** 10.1038/s41598-018-22484-6

**Published:** 2018-03-07

**Authors:** Jia Liu, Guoqiang Jiang, Ping Mao, Jing Zhang, Lin Zhang, Likun Liu, Jia Wang, Lawrence Owusu, Baoyin Ren, Yawei Tang, Weiling Li

**Affiliations:** 1 Department of Biotechnology, Dalian Medical University, Dalian, 116044 Liaoning China; 2 Department of General Surgery, The people’s Hospital of Liaoning Province, Shenyang, 110016 Liaoning China; 3 Academy of Integrative Medicine, Dalian Medical University, Dalian, 116044 China; 4 Department of Biochemistry and Biotechnology, Kwame Nkrumah University of Science and Technology (KNUST), Kumasi, Ghana; 5 Department of Immunology, Dalian Medical University, Dalian, 116044 China

## Abstract

Melanoma is a malignant skin cancer with considerable drug resistance. Increased expression of DNA repair genes have been reported in melanoma, and this contributes to chemotherapy resistance. GADD45A is involved in DNA repair, cell cycle arrest and apoptosis in response to physiologic or environmental stresses. In this study, we investigated the role of GADD45A in chemotherapy response. Firstly, the mRNA expression of profiled DNA repair genes in cisplatin-treated melanoma cells was detected by RT^2^ profiler^TM^ PCR array. We found the expression of GADD45A upregulated in a dose- and time- dependent manner. In addition, suppression of GADD45A sensitized melanoma cells to cisplatin and enhanced cisplatin-induced DNA damage. Flow cytometry revealed that downregulating GADD45A released cells from cisplatin-induced G2/M arrest and increased apoptosis. By using a MEK inhibitor, GADD45A was shown to be regulated by MAPK-ERK pathway following cisplatin treatment. Thus, the induction of GADD45A might play important roles in chemotherapy response in human melanoma cancer and could serve as a novel molecular target for melanoma therapy.

## Introduction

Melanoma, one of the most aggressive and treatment-resistant type of skin cancer, develops from melanocytes, specialized pigmented cells that reside underneath the epidermis^1^. Current treatment strategies for melanoma patients include surgical resection, chemotherapy and radiation therapy^2^. Most early stage melanoma may be cured by surgery. However, treatment of late stage melanoma is still a challenge with increased mortality due to early metastasis and resistance to chemotherapy^3,4^. Therefore, more effective approaches are needed for melanoma patients.

Cisplatin is a DNA-damaging alkylating agent that triggers apoptotic cell death^5^. It is widely used in the treatment of various solid tumors. However, the response rate of cisplatin in melanoma is less than 10% with high recurrence rate due to chemo-resistance^6^. Melanoma cells are actually receptive to the chemotherapeutic drug but they have developed clever escape alternatives to prevent or compensate for the action of the drug^7^. Several reports have described the mechanisms of cisplatin response in melanoma^8,9^. One possible mechanism to counteract the deleterious effects of cisplatin could be hyperaction of DNA repair^10^. A better understanding of the molecular mechanisms of chemo-resistance will give hope for melanoma therapy.

Growth arrest and DNA damage-induced 45 A (GADD45A) belongs to the DNA damage-inducible 45 family which is involved in DNA repair, genomic stability and cell cycle arrest as a result of various physiologic or environmental stresses^11^. GADD45A defective mice exhibited decreased DNA repair and severe genomic instability^12^. It is known to regulate nucleotide excision repair and base excision repair in response to UV radiation^13^. GADD45A is specifically involved in DNA repair, and thus, induce a cell cycle arrest when DNA damage is detected^14^. GADD45 in regulating the cell cycle was observed at the G2/M checkpoint^15^. Cell cycle transitions help cells repair DNA damage and maintain genomic integrity^16^. A previous study has reported that combined Gadd45A and thymidine phosphorylase expression level predicted response and survival of neoadjuvant chemotherapy in gastric cancer^17^. However, whether cisplatin induce GADD45A expression in melanoma cells and its role in chemotherapy response is still unclear.

In the present study, we found that cisplatin treatment elevated the expression of several DNA repair genes, including GADD45A, that may be related to acquired drug response. Inactivation of GADD45A enhanced cisplatin-induced DNA damage, cell cycle arrest and sensitized melanoma cells to cisplatin treatment. In addition, our data showed that cisplatin regulated GADD45A expression through the MAPK-ERK pathway. We demonstrate that GADD45A is a promising target to enhance cisplatin response.

## Results

### Screening of DNA repair genes by RT² Profiler™ PCR Array

**Figure 1:**
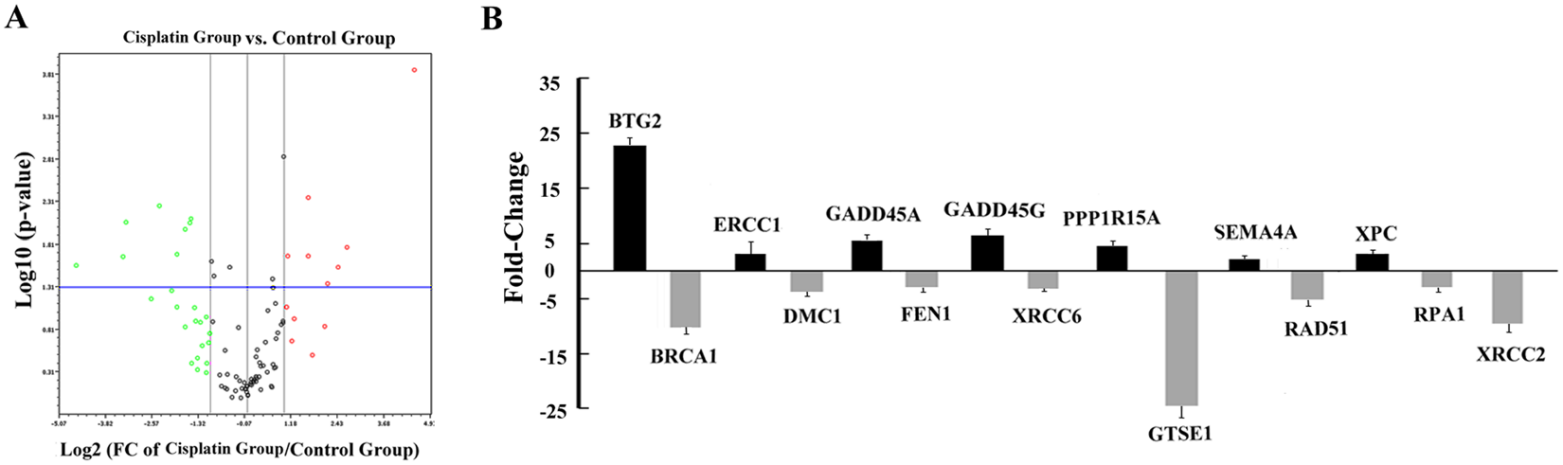
Screening of DNA repair genes by RT² Profiler™ PCR Array. Melanoma A375 cells were treated with cisplatin (4 μ M) for 48 h. RNA isolation and subsequent RT² Profiler™ PCR Array was carried out. (**A**) The Human DNA Damage Signaling RT² Profiler™ PCR Array profile of the expression of 84 genes involved in DNA damage signaling pathways. Genes with fold changes higher than the cut-off value (fold change >2 with p < 0.05) were considered as differentially expressed. Seven genes were up-regulated, whereas eight were down-regulated compared to the controls. (**B**) Histogram showing fold-change relative to the control group following cisplatin treatment of three independent experiments (p < 0.05).

To detect the effects of cisplatin on the regulation of gene expression involved in DNA repair, a Human DNA Damage Signaling RT² Profiler™ PCR Array was used. Figure 1 shows the expression profile of 84 genes involved in the DNA repair pathway in melanoma cells before and after cisplatin treatment (4 μM). The genes with fold changes higher than the cut-off value (fold change >2 with p < 0.05) were selected. Seven transcripts (BTG2, ERCC1, GADD45A, GADD45G, PPP1R15A, SEMA4A and XPC) were upregulated following cisplatin treatment and eight transcripts (BRCA1, DMC1, FEN1, XRCC6, GTSE1, RAD51, RPA1 and XRCC2) were downregulated (Table 1). The induction of ERCC1 by cisplatin leading to refractory chemotherapy response has been discussed in our previous paper^6^. In this study, we focused on exploring the importance of GADD45A induction in melanoma cells post cisplatin treatment.

**Table 1:** Summary of the relative-fold change of genes by RT² Profiler™ PCR Array. Genes with significant (p < 0.05) upregulation or downregulation >2-fold were selected after 48 hours of cisplatin treatment (4 μM).

Gene Symbol	Fold upregulation	p-value	Gene function
BTG2	22.8	0.0001	Other genes involved in DNA repair
ERCC1	3.1	0.0045	Nucleotide excision repair
GADD45A	5.5	0.0292	DNA repair, Cell cycle arrest
GADD45G	6.4	0.0169	Apoptosis
PPP1R15A	4.5	0.0451	Apoptosis
SEMA4A	2.1	0.0213	Damaged DNA Binding
XPC	3.1	0.0215	Nucleotide excision repair
BRCA1	−10.2	0.0217	Damaged DNA Binding, Apoptosis
DMC1	−3.7	0.0205	Damaged DNA Binding
FEN1	−2.9	0.008793	Double-strand Break Repair
XRCC6	−3.2	0.0104	Double-strand Break Repair
GTSE1	−24.5	0.0274	Cell Cycle Arrest
RAD51	−5.2	0.0056	Damaged DNA Binding
RPA1	−2.9	0.0079	Other genes involved in DNA repair
XRCC2	−9.6	0.0086	Damaged DNA Binding

Data from three independent experiments. Statistical analysis was performed using One-Way ANOVA with Bonferroni’s post-hoc test.

### Cisplatin induces GADD45A expression in melanoma cells

**Figure 2:**
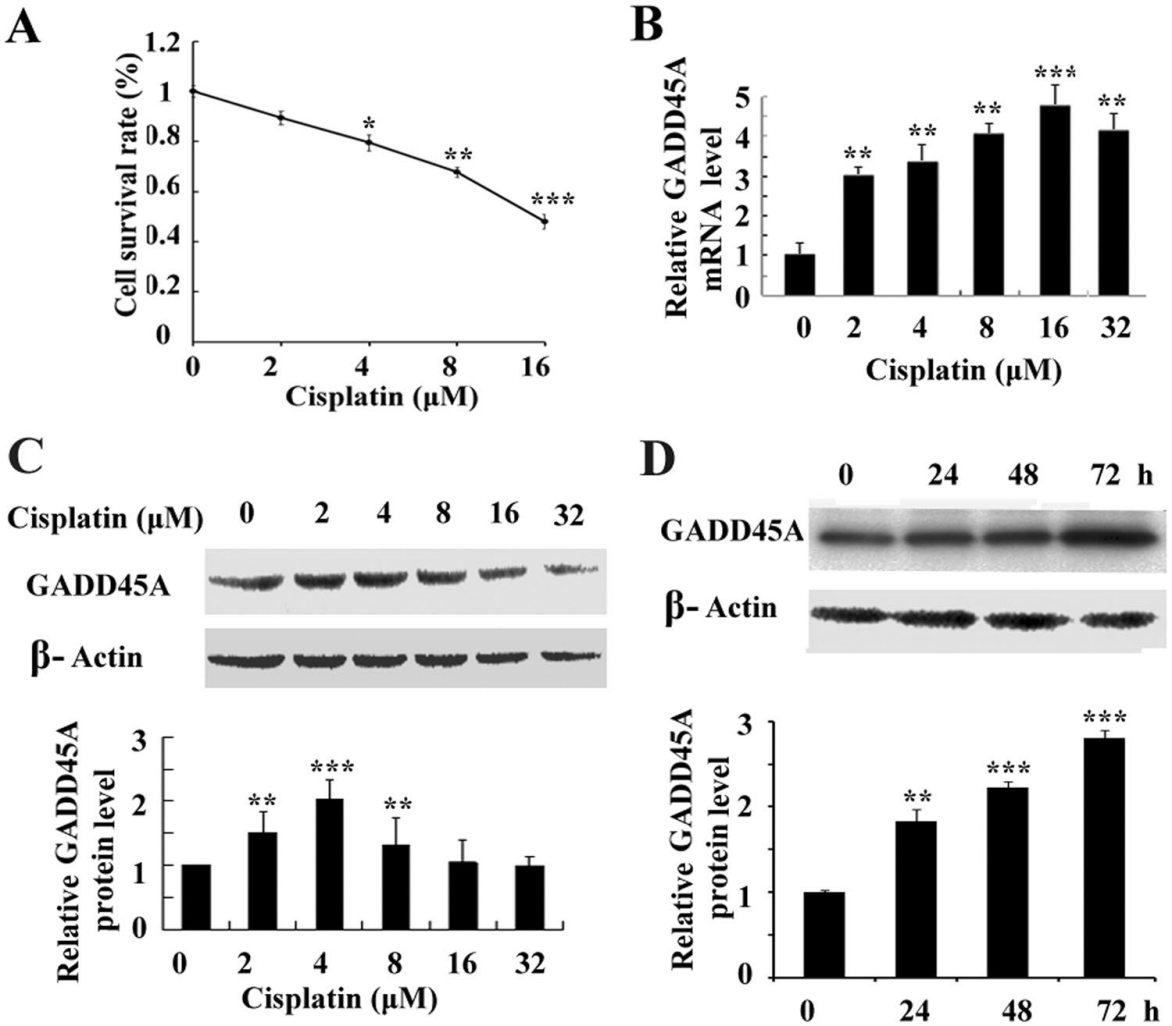
Cisplatin induces the expression of GADD45A in human melanoma cells. (**A**) A375 cells were treated with cisplatin (0, 2, 4, 8 or 16 μM) for 24 h. The viability of the cells was detected by MTT assay. (**B**) A375 cells were treated with cisplatin (0, 2, 4, 8, 16 or 32 μM) for 48 h. Quantitative real-time PCR assay showed mRNA level of GADD45A after cisplatin treatment. (**C**) A375 cells were treated with cisplatin (0, 2, 4, 8, 16 or 32 μM) for 48 h. Western blot showed protein level of GADD45A after cisplatin treatment. (**D**) A375 cells were treated with cisplatin (4 μM) for 24 h, 48 h or 72 h. Western blot showed protein level of GADD45A following cisplatin treatment at different time points. Three independent experiments were performed. Student’ s t-test, *p < 0.05, **p < 0.01, ***p < 0.001 as compared to untreated cells (control).

GADD45A expression post cisplatin treatment was validated by quantitative real-time PCR analysis and western blot analysis. Treatment with Cisplatin (0, 2, 4, 8 or 16 μM) for 24 h induced a reduced proliferation capacity in melanoma cells in a dose-dependent manner (*p* < 0.05). The concentration 4 μM is the IC20 of cisplatin for the cells. Cisplatin treatment (0, 2, 4, 8, 16 or 32 μM) for 48 h significantly induced GADD45A mRNA expression in A375 cells, with the highest expression being 4.9-fold higher as compared to untreated cells (Fig. 2B). Protein expression of GADD45A was also enhanced by cisplatin, peaking at 2.4-fold as compared to untreated cells (Fig. 2C). So we selected and used this cisplatin concentration (4 μM) for subsequent investigations. Our data also indicated a time- dependent increase in the expression of GADD45A following 24 h, 48 h or 72 h cisplatin treatment (4 μM) (Fig. 2D). Altogether, a time- and a dose- dependent increase in GADD45A expression was associated with cisplatin treatment. GADD45A expression may be involved in cisplatin response in melanoma cells.

### Down-regulation of GADD45A sensitized melanoma cells to cisplatin

**Figure 3:**
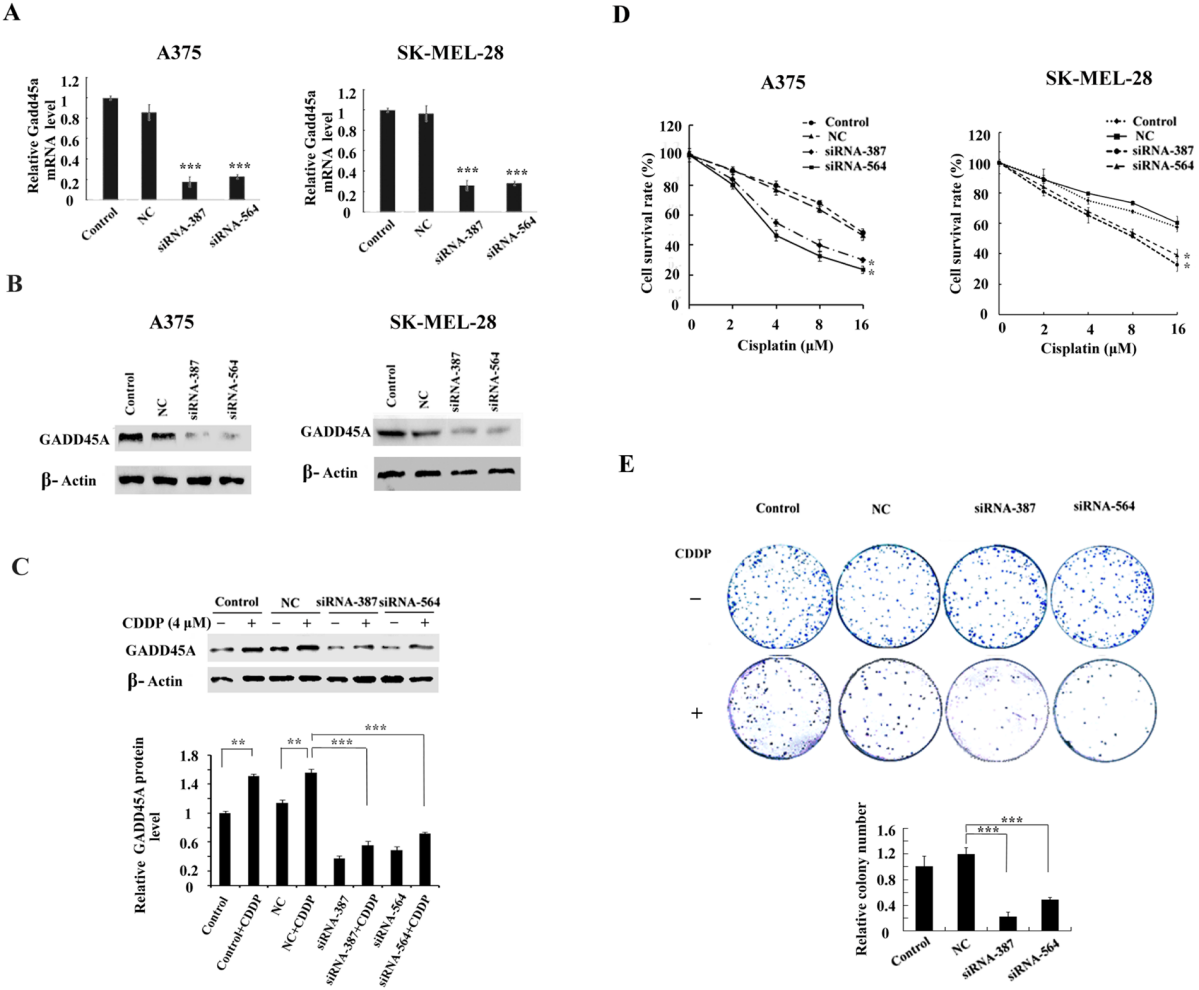
Inactivation of GADD45A impairs the viability and colony formation ability of cisplatin-treated melanoma cells. (A) Quantitative real-time PCR analyzing the mRNA expression of GADD45A in GADD45A-siRNA transfected A375 and SK-MEL-28 cells. (**B**) Western blot analyzed the protein expression of GADD45A in GADD45A-siRNA transfected A375 and SK-MEL-28 cells. (**C**) The protein expression of GADD45A in GADD45A-siRNA transfected and untransfected A375 cells following cisplatin treatment (4 μM, 48 h) by western blot. Protein expressions were normalized to the level of β-actin. (**D**) The cells were treated with cisplatin (0, 2, 4, 8 or 16 μM) for 48 h. The viability of the cells was detected by MTT assay and expressed relative to that of non-transfected cells. (**E**) The cells were treated with cisplatin (2 μM) for 48 h. After 15 days the surviving colonies were stained and counted. The colony numbers were counted, and the colony formation ratio was calculated according to the formula: Colony formation ratio = (number of colonies/number of cells seeded). Histogram shows the colony formation ratio (+SD) of GADD45A-siRNA transfected cells relative to control cells determined from three independent experiments. One-Way ANOVA, *p < 0.05, **p < 0.01, ***p < 0.001.

To further analyze the role of GADD45A in cisplatin response, we transfected melanoma A375 and SK-MEL-28 cells with small interfering RNA (siRNA) of GADD45A: cells were transfected with negative control (NC), GADD45A siRNA-387 or GADD45A siRNA-564. The expression of GADD45A was successfully suppressed by specific siRNA in GADD45A siRNA-transfected cells (Fig. 3A,B). As shown in Fig. 3C, cisplatin increased the expression levels of GADD45A in untransfected control cells (Control) and negative control siRNA-transfected cells (NC). Even if cisplatin treatment caused GADD45A induction also in siRNA transfected cells, GADD45 levels were significantly reduced compared to the negative control group.

Next, we analyzed the chemo-sensitivity of GADD45A siRNA-transfected cells to cisplatin by MTT cell viability assay and colony formation assay. The cell viability of siRNA-387-transfected and siRNA-564-transfected A375 and SK-MEL-28 cells was significantly lower than the Control and NC cells following cisplatin treatment (0, 2, 4, 8 or 16 μM) (Fig. 3D), and this was consistent with the impaired colony formation ability of A375 cells (Fig. 3E). These data suggested that indeed cisplatin-induced GADD45A played a key role in cisplatin-induced treatment refraction in melanoma and that GADD45A inactivation may enhance the chemo-sensitivity of melanoma cells.

### Down-regulation of GADD45A expression increased DNA damage caused by cisplatin

**Figure 4:**
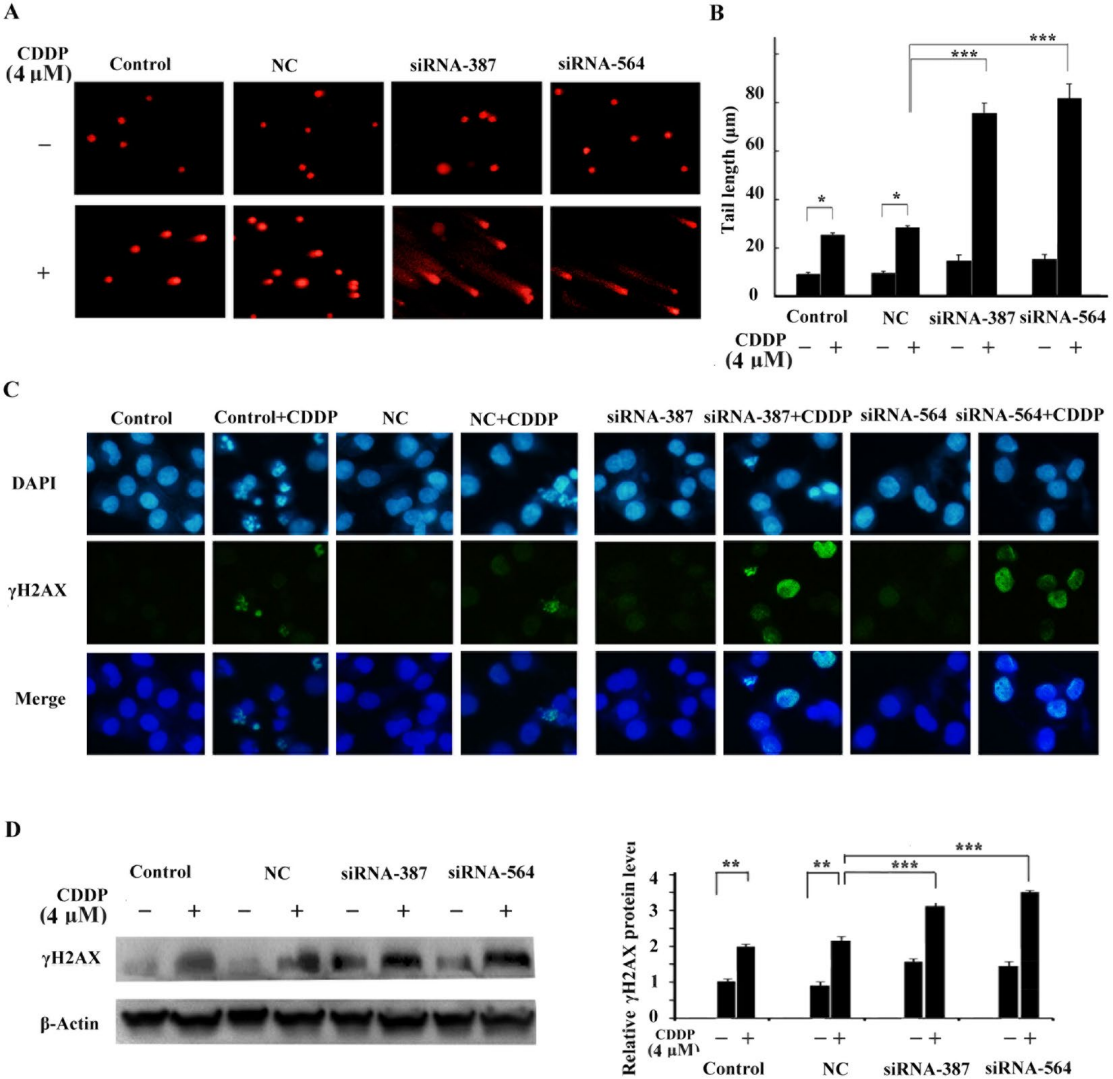
Effect of GADD45A inactivation on cisplatin-induced DNA damage in melanoma cells. (**A**) DNA damage in A375 cells was examined by comet assay. The cells were transfected with negative control siRNA or GADD45A siRNAs. DNA damage was detected following cisplatin treatment (4 μM, 24 h). (**B**) The tail length of the Comet was measured in each cell, expressed in μm as mean ± SD from at least 10 cells in each treatment group. (**C**) DNA damage in A375 cells was examined by Immunofluorescence staining and (**D**) Western blotting. The cells were transfected with negative control siRNA or GADD45A siRNAs, and then examined for γ-H2AX foci formation and expression following cisplatin (4 μM, 24 h) treatment. Student’ s t-test, *p < 0.05, **p < 0.01, ***p < 0.001.

To examine the significance of GADD45A in DNA repair in response to cisplatin, comet assay and immunofluorescence staining were performed following cisplatin treatment (4 μM). As indicated in Fig. 4A, significantly higher DNA damage rate was observed in the GADD45A siRNA-transfected cells than in the untransfected or negative control (NC) cells by comet assay. DNA damage was evaluated by comparing comet “head” and “tail” fluorescence intensity. The tail length of GADD45A siRNA-transfected cells was longer than that of the negative control cells following treatment with cisplatin (Fig. 4B). These data intimated that GADD45A inhibition may influence DNA repair.

Additionally, to further verify whether down-regulation of GADD45A could enhance DNA damage associated with cisplatin treatment in melanoma, we assayed for phosphorylated H2AX (γ-H2AX), an early phase marker of DNA double strand break (DSB). Under normal conditions, the level of γ-H2AX is quite low in mammals, unless DSB is present. Our data showed that inactivation of GADD45A increased γ-H2AX foci formation upon treatment with cisplatin (Fig. 4C). We also detected the expression of γ-H2AX by western blot. The result confirmed that the expression of γ-H2AX in GADD45A siRNA-transfected cells was significantly higher than in the negative control cells following cisplatin treatment (Fig. 4D). Our finding supported the view that GADD45A was a key factor determining melanoma’s sensitivity to cisplatin through modulation of DNA repair.

### Inactivation of GADD45A decreases cisplatin-induced G2/M cell cycle arrest

**Figure 5:**
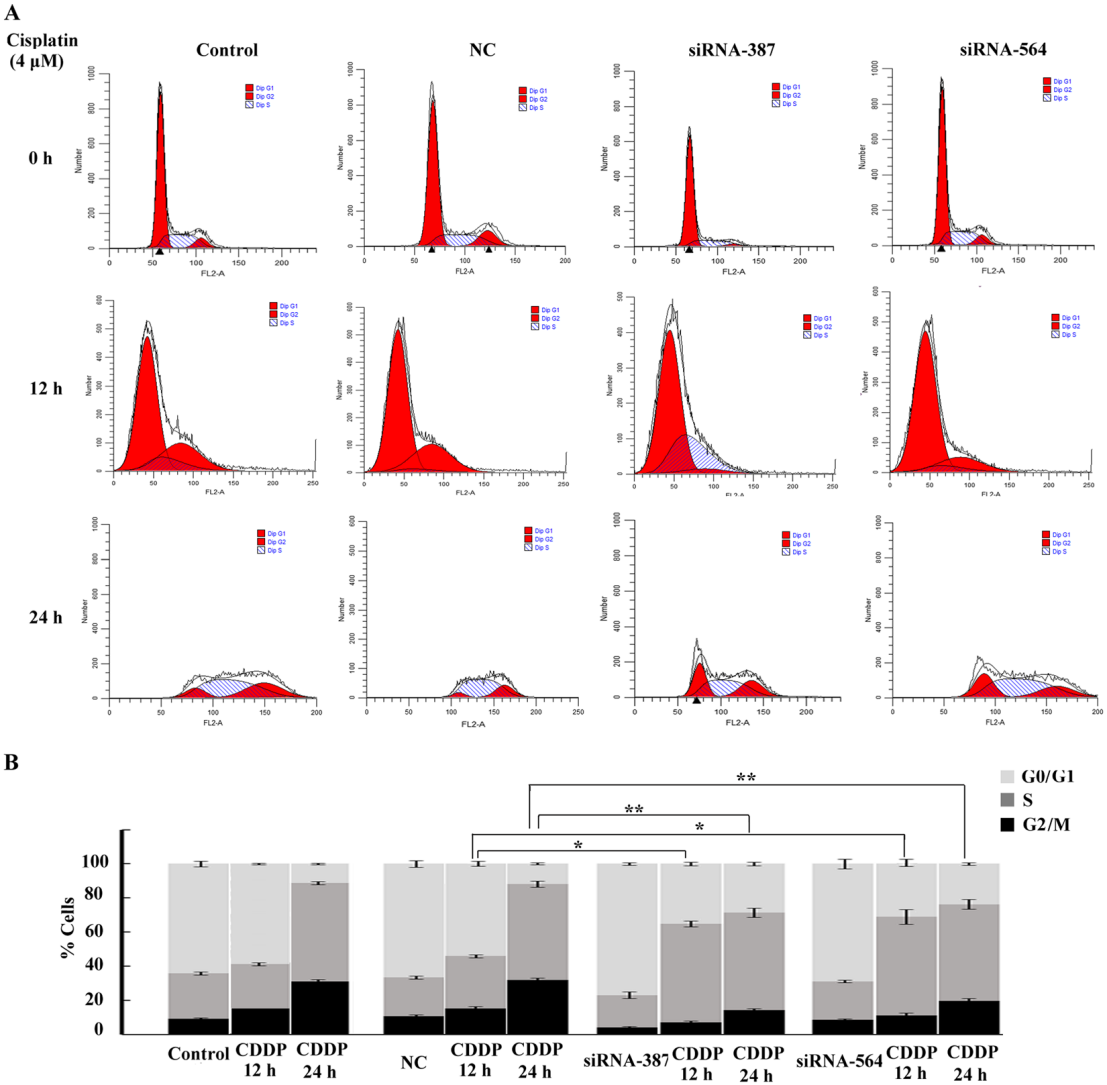
Inactivation of GADD45A decreased cisplatin-induced G2/M cell cycle arrest. (**A**) The cell cycle distribution was analyzed using flow cytometry following cisplatin treatment (4 μM) for 0 h, 12 h and 24 h. The images are representative of three independent experiments. (**B**) Stacked bar graph indicating the percentage of cells at various stages of the cell cycle. The percentage of G2/M phase cells was significantly decreased in the GADD45A-transfected cells following cisplatin treatment. Three independent experiments were performed. Student’ s t-test, *p < 0.05, **p < 0.01, ***p < 0.001.

Since GADD45A inhibition could induce DNA damage and the DNA damage was related to cell cycle arrest^18^, we detected the role of GADD45A in the cell cycle of melanoma A375 cells under cisplatin stimulation by flow cytometry. The percentage of G2/M phase cells increased to 16.2 ± 1.7% following cisplatin treatment (4 μM) for 12 h in the negative control cells. However, the percentage of G2/M phase cells decreased to 7.3 ± 1.8% and 9.1 ± 1.4% in GADD45A siRNA-387- and siRNA-564-transfected cells, respectively, under the same treatment conditions. At 24 h post cisplatin treatment (4 μM), the percentage of G2/M phase cells increased to 37.8 ± 2.1% in the negative control cells while the percentage of G2/M phase cells were 12.2 ± 2.3% and 19.7 ± 2.4% in the GADD45 A siRNA-387- and siRNA-564-transfected cells, respectively. Similar to other studies, we found that cisplatin treatment caused G2/M arrest of A375 cells in the cell cycle and more intriguingly that inactivation of GADD45A could eliminate this phenomenon (Fig. 5A,B).

### GADD45A protects melanoma cells from cisplatin-induced apoptosis

**Figure 6:**
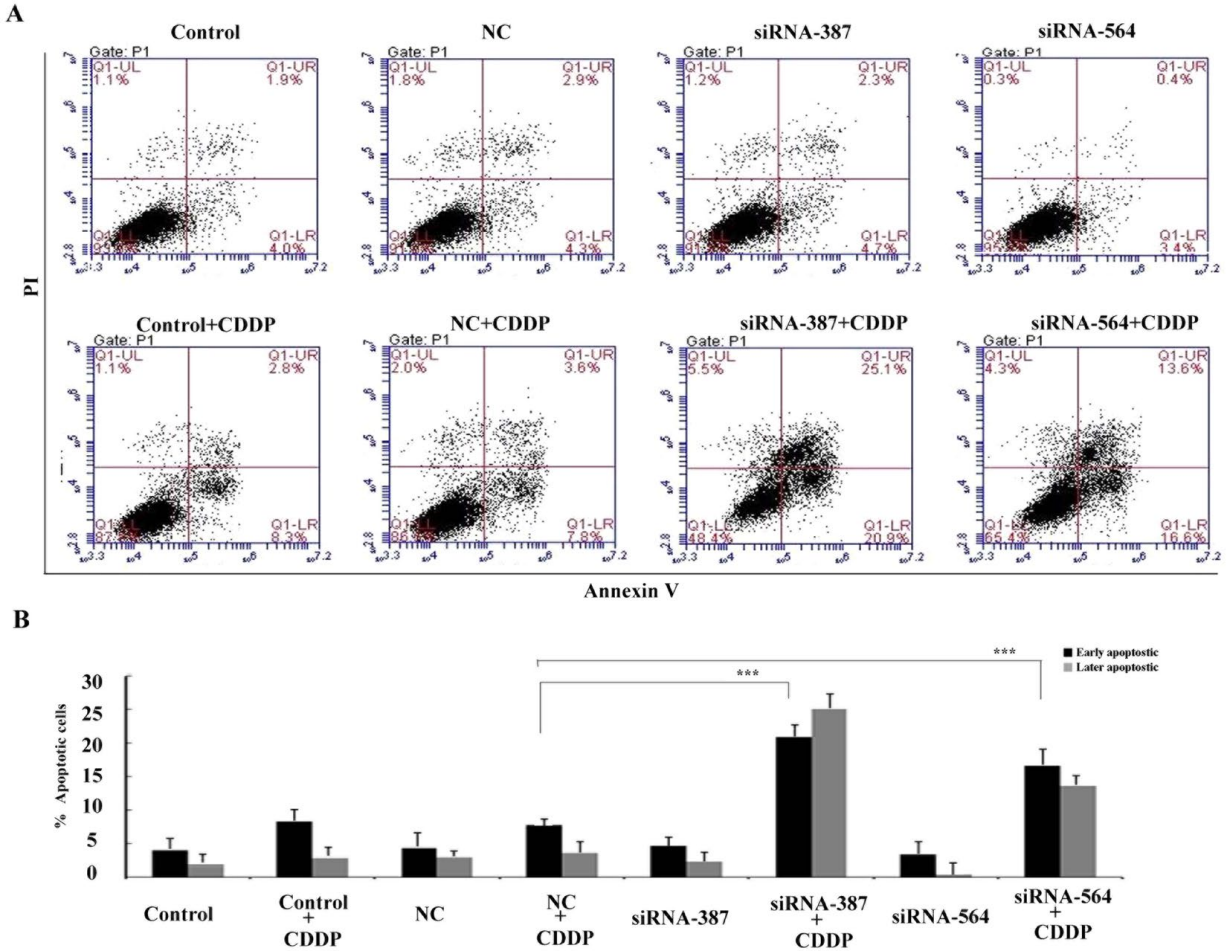
Suppression of GADD45A increased cisplatin-induced cell apoptosis. (**A**) Annexin V/PI staining flow cytometry was performed to detect apoptosis. The lower right quadrant of the fluorescence activated cell sorting (FACS) indicates the percentage of early apoptotic cells (Annexin V-stained cells), while the upper right quadrant indicates the percentage of late apoptotic cells (Annexin V+/PI-stained cells). The cell distribution was analyzed using flow cytometry after cisplatin treatment (4 μM, 24 h). The images are representative of three independent experiments. (**B**) Histogram showing the percentage of early and late apoptotic A375 cells after treatment with 4 μM cisplatin for 24 h. Student’ s t-test, *p < 0.05, **p < 0.01, ***p < 0.001.

To evaluate if GADD45A chemo-response effect on A375 cells was apoptosis related, flow cytometry was used to detect Annexin V-PI labelled cells (Fig. 6A). The early stage apoptotic cell populations in GADD45A inactivated cells (siRNA-387- and siRNA-564) treated with 4 μM cisplatin for 24 h were 21.7 ± 2.32% and 17.2 ± 3.1%, respectively, as compared to 8.6 ± 1.2% in the negative control (NC) cells treated with 4 μM cisplatin (Fig. 6B). The siRNAs for GADD45A alone did not influence apoptosis induction. These data suggested that GADD45A was involved in cisplatin-induced apoptosis. Inactivation of GADD45A could release melanoma cells from cell cycle arrest and enhance apoptosis.

### The MAPK pathway regulates GADD45A protein levels following cisplatin treatment in melanoma cells

**Figure 7:**
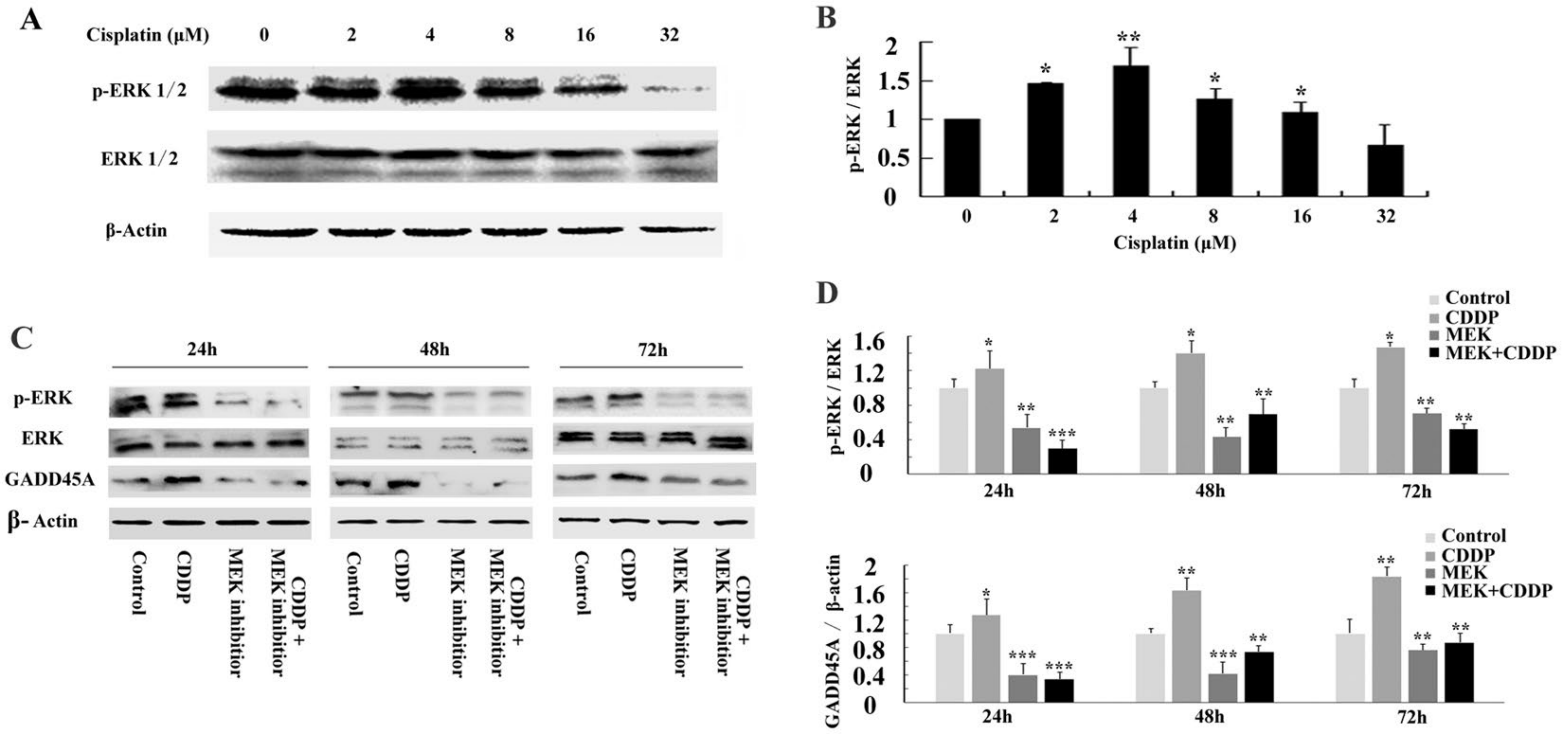
GADD45A protein induction by cisplatin is regulated by the MAPK-ERK pathway in human melanoma cells. (**A**) Western blots showing protein levels of p-ERK1/2 and ERK1/2 in A375 cells treated with cisplatin (0, 2, 4, 8, 16 or 32 μM) for 48 h. (**B**) Histogram showing mean level of ERK1/2 activation (p-ERK1/2/total ERK1/2) from three independent experiments after cisplatin treatment. (**C**) Western blots showing protein levels of p-ERK1/2, ERK1/2 and GADD45A in A375 cells treated with cisplatin (4 μM), PD0325901 (1 μM), or both cisplatin and PD0325901 for 24 h, 48 h or 72 h. (**D**) Histogram showing mean level of ERK1/2 activation (p-ERK1/2/total ERK1/2) and GADD45A expression from three independent experiments following cisplatin treatment. Student’ s t-test, *p < 0.05, **p < 0.01, ***p < 0.001 for CDDP vs Control or MEK inhibitor vs Control or CDDP + MEK inhibitor vs CDDP.

To further investigate the molecular mechanisms regulating GADD45A by cisplatin, we probed the MAPK-ERK pathway. Cisplatin treatment caused a dose-dependent activation of ERK1/2 (Fig. 7A,B). An inhibitor of MEK1/2, PD0325901(Selleck, China), was used to determine the necessity of ERK activation in the induction of GADD45A by cisplatin. A375 cells were treated with either cisplatin (4 μM), PD0325901 (1 μM), or both for 24 h, 48 h or 72 h. The cell lysates were obtained to determine the levels of p-ERK1/2 and GADD45A. PD0325901 successfully inhibited the activation of ERK caused by cisplatin. More importantly, PD0325901 prevented the cisplatin-induced increase in GADD45A expression (Fig. 7C,D). It suggested that MAPK-ERK activation may be critical in regulating the expression of GADD45A following cisplatin treatment in melanoma cells.

## Discussion

The incidence of melanoma has been increasing faster than any other cancer, with a 2.6% annual increase worldwide over the last decade^19^. The ineffectiveness of surgical resection or chemotherapy coupled with recurrence makes it urgent to explore molecular targets associated with melanoma progression or chemotherapy resistance to develop novel therapies. In this study, we found that cisplatin dramatically induced the expression of GADD45A in melanoma and that inhibition of GADD45A sensitized melanoma cells to cisplatin. It suggested that GADD45A could be involved in cisplatin response and may be a new target for melanoma treatment.

GADD45A is known to regulate DNA repair in response to DNA damage^20^. Overexpression of GADD45A can stimulate nucleotide excision repair, whereas loss of GADD45A expression in lymphoblasts resulted in a substantially reduced nucleotide excision repair activity^21^. GADD45A deficiency has also been reported and linked to reduced base excision repair and decreased APE interaction with PCNA^22^. Previous study showed that GADD45A could be induced by UV radiation to increase local DNA accessibility that is involved in DNA repair^23^. GADD45A induction also protected HepG2 cells from UV radiation-induced DNA damage^24^. Lack of GADD45A induction in cervical carcinomas correlated with a good clinical response to radiotherapy^25^. In this study, we showed that cisplatin treatment stimulated GADD45A expression. Downregulation of GADD45A sensitized melanoma cells to cisplatin and dramatically increased DNA double strand break. The expression of GADD45A in melanoma might increase DNA repair and thus protect melanoma from cisplatin-induced DNA damage.

Cancer cells can avert apoptosis and survive through DNA repair when held for prolonged period at the G2/M phase of the cell cycle^26^. Some molecular changes have been suggested to liberate cells from G2/M phase arrest to go through DNA damaging agents-induced apoptosis^27,28^. A previous report described GADD45A inhibited Cdc2 kinase and induced G2/M arrest^29^. Our data showed that cisplatin stimulated the expression of GADD45A in melanoma cells to cause enhanced G2/M arrest. Such arrest helped cells to repair DNA damage and prevented melanoma cells from undergoing apoptosis. On the contrary, suppression of GADD45A released the cells from G2/M arrest to significantly boost cisplatin-induced apoptosis of melanoma cells. Therefore, melanoma cells may evade cisplatin-induced apoptosis by accumulating cells in G2/M phase of the cell cycle to allow repair of damaged DNA.

The role of GADD45A in apoptosis is controversial^30^. GADD45A has been associated with apoptosis after oncogenic and genotoxic stresses^31^. In some study, GADD45A expression was up regulated by genotoxic agents and acted as a pro-apoptotic protein^32^. While in other studies, GADD45A was described to contribute to cell survival^14^. Our data suggested GADD45A induction by cisplatin could arrest cell cycle and trigger DNA repair to overcome apoptosis. However, down-regulating GADD45A increased the percentage of apoptotic cells following cisplatin treatment. Altogether, these results demonstrated that GADD45A inhibition was able to sensitize melanoma cells to cisplatin-induced apoptosis *in-vitro*. However, we admit the limitation of the absence of animal models in this study, and thus our exploratory results need further investigation.

Mitogen-activated protein kinase (MAPK) has been reported to be responsible for chemo-resistance^33^. Wang and colleagues have recently demonstrated that activation of MAPK-ERK pathway is involved in regulating the UV induction of GADD45A promoter^34^. Study showed that DNA-damaging agents, such as cisplatin, activated ERK^35^. This was also confirmed in our study that the treatment of melanoma cells with cisplatin triggered ERK activation. More interestingly, inhibition of the MAPK-ERK pathway by MEK inhibitor prevented cisplatin-induced GADD45A upregulation. It was suggested that MAPK-ERK pathway might be involved in the regulation of GADD45A by cisplatin treatment.

In conclusion, our data suggested that GADD45A might play important roles in cisplatin response. Suppression of GADD45A engendered enhanced chemo-sensitivity. Thus, GADD45A may serve as a novel molecular therapeutic target for malignant melanoma cancer.

## Materials and Methods

### Materials and reagents

The apoptosis detection kit, cell cycle detection kit and comet assay kit were bought from KeyGen Biotech company (Nanjing, China). MTT (Thiazolyl blue tetrazolium bromide) and DAPI (DAPI, dihydrochloride) were obtained from Sigma Chemical Co. (MO, USA). Antibodies against GADD45A, ERK, p-ERK and β-actin were obtained from Proteintech Group Inc. (Chicago, USA). Antibodies against γH2AX was from Cell Signaling Technology (MA, USA) while the enhanced chemiluminescence (ECL) kit was purchased from Amersham Life Science, Inc. (United States). A specific inhibitor of MEK1/2 (PD0325901) was purchased from Selleck (Shanghai, China).

### Cell lines and cell culture

The melanoma A375 and SK-MEL-28 cell lines were bought from American Type Culture Collection (ATCC). Cells were grown in 25 cm cell culture flask and incubated at 37 °C with 5% CO_2_ in DMEM medium (Gibco, NY, USA) containing 10% fetal bovine serum (FBS) with 100 U/ml penicillin (Gibco, NY, USA) in a humidified incubator.

### RT^2^ Profiler PCR Array

The relative mRNA expressions of genes involved in DNA damage activities were analyzed following cisplatin stimulation of A375 using an RT^2^ ProfilerTM PCR array (Human DNA Damage Signaling Pathway, Qiagen) in adherence to manufacturer’s instructions^36^. In brief, cells treated with or without cisplatin (4 μM) were incubated at 37 °C under a humidified atmosphere containing 5% CO_2_. After incubation, washing cells with PBS, total RNA was extracted using RNeasy Mini Kit (Qiagen), and cDNA was synthesized by RT-PCR using an RT^2^ First Strand Kit (Qiagen) according to the manufacturer’s protocol. The cDNA was applied to the Profiler PCR array and real-time PCR was performed on a Sequence Detection System (ABI PRISM^®^ 7000; Life Technologies Corporation) using PCR master mix (SA Biosciences RT^2^ qPCR Master Mix; Qiagen) for SYBR Green detection for each reaction. Samples were amplified with a precycling hold at 95 °C for 5 min, followed by 40 cycles of denaturation at 95 °C for 15 s and annealing for 1 min at 60 °C. Changes in mRNA expression were analyzed using ∆∆Ct method. The expression levels were quantified relative to the values obtained for some housekeeping genes (ACTB, B2M, GAPDH, HPRT1, and RPL13A).

### RNA extraction and reverse transcription

Cells were planted at 5000 cells/well in a 24-well plate. After incubation, washing cells with PBS, total RNA was extracted using Trizol regent (Life technologies, California) from cells according to the protocols of the manufacturer. cDNA for quantitative real-time PCR was synthesized using a High-capacity cDNA Reverse Transcription Kit by the manufacturer’s instructions.

### Real time quantitative PCR analysis

RT-PCR analysis was performed to quantify the cDNAs of GADD45A in an Applied Biosystems 7900HT Fast RT-PCR System using Taqman® Gene Expression Assay^37^. Briefly, reaction mixtures were prepared in 20 μl volume consisting of 10 ul 20X Taqman Gene Expression Master Mix, 1 ul specific 20X Taqman Gene Expression Assay, 4 ul cDNA template (10–100 ng), and 5 ul RNase-free water. Routine real time qPCR conditions were: Enzyme activation at 95 °C for 10 min, followed by 40 cycles of DNA denaturation at 95 °C for 10 min, primer annealing and DNA polymerase extension at 60 °C for 60 sec. Sequences of RT-PCR primers for GADD45A transcript are listed as follows: 5′-CTGGGAATTTGGCGACGTAA-3′ and 5′-ATGGATGTAGTCTGGGTGCAG-3′. The quantification of each PCR product was performed by 2^−ΔΔCt^ method^38^.

### RNA interference

The siRNAs for GADD45A were purchased from GenePharma (Shanghai, China). The siRNA was transfected with Lipofectamine^®^ 2000 Transfection Reagent (Life Technologies Corporation) in accordance with manufacturer’s instructions. GADD45A siRNA sequences are: GADD45A siRNA-387 (5′-GGAGGAAGUGCUAGCAAATT-3′), GADD45A siRNA-564 (5′-GCGAGAACGACAUCAACAUTT-3′).

### MTT assay

MTT assay was used to assess the viability of cells following cisplatin treatment. Briefly, using a 96-well plate, cells were plated at a density of 10^4^ cells/well and incubated for 24 h. After cells were either transfected with siRNA or treated with cisplatin, MTT solution (20 μl; 5 mg/ml in PBS) was added to individual wells and incubated for 4 h at 37 °C. Following incubation, residual MTT solution was discarded and the crystals were resolved with 150 μl DMSO. Using a plate reader (Multiskan Ascent, Thermo Scientific, USA), absorbance was measured at 540 nm for each well.

### Colony formation assay

For colony formation assays, about 500 cells were plated in a 35 mm dish. Then 1 ml medium with or without cisplatin (2 μM) was added into each well. On day 15 post treatment, 70% ethanol was used to fix the cells for 5 min, followed by staining with 10% ethanol crystal violet (Sigma, MO, USA) solution. The number of colonies formed were counted.

### Comet assay for DNA damage analysis

DNA damage analysis by cisplatin treatment in melanoma cells was determined using the Comet assay. Briefly, cells were treated with or without cisplatin (4 μM) for 24 h in complete medium, harvested and suspended in ice-cold PBS buffer. A volume of 75 μl low-melting-point agarose (0.5% (w/v) containing approximately 1 × 10^4^ cells was pipetted onto a 1.0% (w/v) agarose-coated frosted glass slide, covered with a coverslip and placed on ice for 10 mins to set. Afterwards, the coverslip was removed and 1.0 (w/v) agarose was used to form a thin layer to cover the slide. The slide was horizontally placed in an electrophoresis tank containing buffer after immersing in ice-cold lysis solution for 2 h and subjected to electrophoresis at 30 V at 4 °C for 30 mins. A 2.5 μg/ml PI solution was used to stain the cells for 5 mins and subsequently observed at 200X magnification using a microscope. Tail length was quantified by measurement from the center of the nucleus to the tail end of a cell.

### Immunofluorescence staining

Prior to cisplatin treatment (4 μM), cells were seeded on glass coverslips (Fisher Scientific). Following treatment, 4% paraformaldehyde was used to fix the cells, washed using PBS and permeabilized with 0.2% Triton X-100. The cells were subsequently incubated in a blocking buffer composed of 3% bovine serum albumin in PBS, followed by overnight incubation with anti-γH2AX antibodies at 4 °C. Biotinylated horse anti-goat IgG antibody (Vector Labs) was added and the resulting mixtures were incubated with the appropriate fluorescent-conjugated secondary antibody [Alexa Fluor 488 (Molecular Probes, 1:500) and Alexa Fluor 594 goat anti-mouse (Molecular Probes, 1:500)]. Cells were counterstained with DAPI (Sigma Aldrich). Using a fluorescent mounting medium (Vector Labs), coverslips were placed on the slides. Slides were mounted on a confocal microscope (Carl Zeiss, LSM 710) for observation. Fluorescence intensity was quantified using Image J software (NIH, MD, USA). For foci quantification, cells with more than 10 foci were counted as positive according to standard procedure.

### Cell cycle assessment using flow cytometry

Progression of cells through the cell cycle was assayed by measuring DNA fragmentation with PI staining. Following 24 h treatment with cisplatin (4 μM), the cells were rinsed twice with PBS and then fixed overnight in 70% ethanol at 4 °C. PI dissolved in PBS containing RNase was used to stain the DNA for 1 h at 37 °C. The cells were sorted using Accuri C6 flow cytometer (BD Biosciences, USA) and the data were subsequently analyzed using ModFitLT V4.1.

### Apoptosis assessment by flow cytometry

Apoptosis was evaluated using Annexin V-FITC assay kit following the protocol of the manufacturer. Briefly, after a 24 h treatment with 4 μM cisplatin, the A375 cells were harvested, washed thoroughly in PBS, and treated with Annexin V-FITC and PI away from light for 10 min. Accuri C6 flow cytometer (BD Biosciences, USA) was used to sort and analyze the stained cells.

### Western blot

RIPA buffer (Beyotime, China) supplemented with 0.01% PMSF (Biocolors, China) was used for total protein extraction from the cells. Equal protein concentration was loaded and separated by electrophoresis on a 10% sodium dodecyl sulfate polyacrylamide gel electrophoresis gel then then blotted onto polyvinylidene difluoride membranes from Bio-Rad, USA. At room temperature, the membranes were incubated in 5% skimmed milk for 1 h. The appropriate primary antibodies at optimal concentrations were applied to the membranes in blocking buffer and kept at 4 °C overnight. Species specific secondary antibodies were added onto the membranes at room temperature for 1 h after washing the membranes to remove unbound primary antibodies. Membranes were thoroughly washed in PBS. The protein bands were visualized using an ECL kit (Amersham Life Sciences, USA). ChemiDoc XRS image analyzer (Bio-Rad, USA) was used for image capturing. Image J software (NIH, MD, USA) was used to quantitatively analyze the protein expression levels. β-actin protein expression served as the internal reference.

### Statistical analysis

All experiments were independently repeated thrice. Data are presented as means ± SD. Student’s *t*-test or One-Way ANOVA was used for assessing statistical differences, where appropriate, using Prism 5.0 (GraphPad Software Inc., CA, USA). Statistical significance was generally indicated at *p*-value less than 0.05.
